# Plant-specific microbial diversity facilitates functional redundancy at the soil-root interface

**DOI:** 10.1007/s11104-024-07097-5

**Published:** 2024-12-05

**Authors:** Wisnu Adi Wicaksono, Martina Köberl, Richard Allen White, Janet K. Jansson, Christer Jansson, Tomislav Cernava, Gabriele Berg

**Affiliations:** 1 Institute of Environmental Biotechnology, Graz University of Technology, Graz, Austria; 2 Earth and Biological Sciences Division, Pacific Northwest National Laboratory, Richland, WA USA; 3 Department of Bioinformatics and Genomics, North Carolina Research Campus (NCRC), The University of North Carolina at Charlotte, Kannapolis, NC USA; 4 Computational Intelligence to Predict Health and Environmental Risks (CIPHER), Department of Bioinformatics and Genomics, The University of North Carolina at Charlotte, Charlotte, NC USA; 5 Environmental Molecular Sciences Laboratory, Pacific Northwest National Laboratory, Richland, WA USA

**Keywords:** Medicinal plants, Microbiome, Rhizosphere, Amplicon sequencing, Metagenome

## Abstract

**Aims:**

Plant-specific microbial diversity reflecting host-microbe coevolution was frequently shown at the structural level but less on the functional scale. We studied the microbiome of three compartments at the soil root interface (root endosphere, rhizosphere, bulk soil) of medicinal plants cultivated under organic management in Egypt. The study aimed to examine the impact of the rhizosphere on microbial community composition and diversity in desert agricultural soil, as well as to identify specific functions associated with the rhizosphere.

**Methods:**

The microbiome community structure, diversity, and microbial functioning were evaluated through the utilization of 16S rRNA gene amplicon and shotgun metagenome sequencing.

**Results:**

We found the typical rhizosphere effect and plant-species-specific enrichment of bacterial diversity. The annual plants *Calendula officinalis* and *Matricaria chamomilla* (*Asteraceae*) were more similar than the perennial *Solanum distichum* (*Solanaceae*). Altogether, plant species explained 50.5% of the variation in bacterial community structures in the rhizosphere. Our results indicate a stronger effect of the plant species in terms of modulating bacterial community structures in the rhizosphere than in root endosphere samples. The plant-driven rhizosphere effect could be linked to redundant plant beneficial functions in the microbiome, while enrichment of specific genes related to amino acid ion transport and metabolism, carbohydrate transport and metabolism, defense mechanisms, and secondary metabolites biosynthesis were more specific.

**Conclusions:**

The study explores the microbiome continuum at the soil-root interface of medicinal plant species, revealing significant bacterial community structure shifts and plant specificity. The study provides insights into the essential microbiome components contributing to rhizosphere functionality.

**Supplementary information:**

The online version contains supplementary material available at 10.1007/s11104-024-07097-5.

## Introduction

Plants and their associated microbes form functional assemblages of species that are referred to as holobionts (Cordovez et al. 2019; Vandenkoornhuyse et al. 2015). The plant microbiota is mainly assembled from seed and soil microorganisms and forms distinct microbial communities in each plant compartment (Abdelfattah et al. 2022; Berg and Raaijmakers 2018; Kusstatscher et al. 2021). The soil-root interface, also known as the rhizosphere, is a hotspot for plant-microbe interactions with high microbial abundances and activity due to the high nutrient content (Bakker et al. 2013; Berendsen et al. 2012; de la Fuente Cantó et al. 2020). The ‘rhizosphere effect’ affects an interwoven network of all domains of life interacting and providing important ecosystem services (Rovira 1956; White et al. 2017). Specific plant-microbe networks have likely emerged from co-evolution (Chaudhry et al. 2021; Delaux and Schornack 2021; Wassermann et al. 2019; Wicaksono et al. 2021), and were shaped by domestication (Abdelfattah et al. 2021; Bulgarelli et al. 2013; Pérez-Jaramillo et al. 2016).

The composition of the plant microbiome is to a certain degree plant species-specific; this degree depends on many factors, e.g., host morphology and secondary metabolism. Cultivation-based studies together with cultivation-independent community fingerprinting methods showed a significant impact of the plant genotype and soil type on the plant microbiome (Berg et al. 2006; Berg and Smalla 2009; Weinert et al. 2010). This impact was later confirmed and disentangled in detail by studies using model plants i.e., *Lotus japonicus* and *Arabidopsis thaliana* as well as non-model plants i.e., *Solanum lycopersicum*,* Sorghum bicolor*, and *Populus trichocarpa* based on high-throughput sequencing techniques (Bulgarelli et al. 2012; Cordovez et al. 2021; Deng et al. 2021; Veach et al. 2019; Wippel et al. 2021). Plant phylogeny is often linked to specific groups of associated microorganisms, e.g., *Brassicaceae* plants have no mycorrhiza and enrich specific bacterial communities driven by glycosinolates (Sharma et al. 2023), while legumes mainly interact with *Rhizobiales* (Tsiknia et al. 2021). However, less is known about functional diversity in the rhizosphere and its plant specificity.

The plant microbiota contributes to resilience against biotic and abiotic stress and triggers important processes in plants, e.g., germination and ripening, circadian and annual cycles, cold acclimation, and fruit and seed formation (Berg et al. 2016; Schlaeppi and Bulgarelli 2015). Moreover, the rhizosphere microbiota contributes to soil ecosystem functioning as well as to biogeochemical cycling (Philippot et al. 2013). For example, soil microorganisms play a crucial role in the cycling of soil carbon by decomposing organic matter and facilitating its transformation through various metabolic pathways (Wu et al. 2023). This process contributes to stabilizing organic carbon and influences soil carbon storage and turnover. Previous studies have indicated that the resistance of microbial communities to pathogen invasion is linked to their level of structural diversity (Berg et al. 2017; Kinnunen et al. 2016; Mallon et al. 2015). However, the specificity of functional diversity, and whether it correlates with structural diversity of microbial communities remains mostly unexplored. We hypothesized that structural diversity is higher in plant microbiomes than functional diversity and that even plants with a specific microbial community show similar microbiome functions.

The present study assessed the relationship between structural and functional diversity with different model plants. The study aimed to understand the rhizosphere effect on microbial community composition and diversity in desert farm soil and identify distinctive rhizosphere functions. We selected three medicinal plants namely: German chamomile (*Matricaria chamomilla* L.), marigold (*Calendula officinalis* L.), and nightshade (*Solanum distichum* Schumach. and Thonn.). These selected medicinal plants are among the predominant herbs in Sekem. Additionally, they are recognized for their antimicrobial properties and bioactive constituents. (Köberl et al. 2011, 2016, 2019). Plants were cultivated in desert soil under organic (biodynamic) management at the desert farm Sekem in Egypt. We conducted 16S rRNA gene fragment-based community profiling and deep shotgun metagenomic sequencing with bulk desert farm soil, and rhizosphere soils as well as root endosphere samples of the aforementioned plant species. In this study, we aimed to address the following questions: (1) How does the rhizosphere effect influence the composition and diversity of microbial communities in desert farm soil? (2) Are there core functions that are distinctive to rhizosphere communities within the desert agro-ecosystem? (3) Are plant-specific rhizobacteria, as represented by metagenome-assembled genomes (MAGs), equipped with multifunctional capabilities? Additionally, (4) do these taxa have the capacity to degrade and cycle sequestered carbon?

## Materials and methods

### Experimental design and sampling

All samples were obtained from the organically managed Sekem farm Adleya (www.sekem.com) located in the north-eastern desert region of Egypt near Bilbeis (30°13’44"N, 31°23’39"E) at Sharqia governorate. The soil texture at the desert farm was classified as loamy sand, with 4% clay and 13% silt, an organic carbon content of 0.8%, and an alkaline pH of 8.4 (Luske and van der Kamp 2009). The three medicinal plants (*Matricaria chamomilla* L., *Calendula officinalis* L., and *Solanum distichum* Schumach. and Thonn.) were grown in close proximity under field conditions. At the time of sample collection, all medicinal plants were in the flowering stage. Rhizosphere and root endosphere samples of the medicinal plants were analyzed and compared to bulk soil. From each plant species, four independent composite samples were taken, each consisting of 5–10 plant roots with adhering soil. Four composite samples of bulk soil were collected in a horizon of 10–30 cm depth. The detailed sampling procedure and plant cultivation conditions are described by Köberl et al. (2011).

## Total DNA extraction

To isolate total community DNA from the soil and rhizosphere, 5 g of soil or roots with adhering soil were added to 45 ml of sterile 0.85% NaCl and vortexed. For isolation from the root endosphere, 5 g of roots were surface sterilized with 4% NaOCl for 5 min. The roots were washed three times with sterile distilled water, and then 10 ml of sterile 0.85% NaCl were added and homogenized using a mortar and pestle. For isolation of total DNA from the rhizosphere, root endosphere, and soil, 4 ml of the suspensions were centrifuged (16,000 × *g*, 4 °C) for 20 min and the resulting microbial pellets were stored at −70 °C. Total community DNA was extracted using the FastDNA SPIN Kit for Soil (MP Biomedicals, Solon, OH, USA) and quantified using a NanoDrop 2000c spectrophotometer (Thermo Scientific, Waltham, MA, USA). Metagenomic DNA samples were encoded using abbreviations indicating: (1) soil or plant species (S = agricultural soil, Mc = *Matricaria chamomilla*, Co = *Calendula officinalis*, Sd = *Solanum distichum*), (2) independent replicate sample (1–4), and (3) compartment (R = rhizosphere, Re = root endosphere, the soil has no further designation).

## Bacterial community analysis by 16S rRNA gene fragment profiling

To obtain a comprehensive overview of the total bacterial community structure, universal primers were used in combination with PNA PCR blockers to reduce the co-amplification of chloroplasts and mitochondrial DNA (Lundberg et al. 2013). The hypervariable V4 region of the 16S rRNA gene was amplified with the primer pair 515 F/806R (Caporaso et al. 2012), which carried sample-specific tags. The reaction mixture for the PCR (30 µl) contained 1× Taq-&GO (MP Biomedicals, Solon, OH, USA), 0.2 µM of each primer, 0.75 µM of pPNA and mPNA (PNA Bio, Thousand Oaks, CA, USA), respectively, and 2 µl of template DNA dilution (96 °C, 5 min; 30 cycles of 96 °C, 1 min; 78 °C, 5 s for PNA annealing; 54 °C, 1 min; 74 °C, 1 min; and elongation at 74 °C, 10 min). PCR products of three independent reactions were pooled in equal volumes and purified by employing the Wizard SV Gel and PCR Clean-Up System (Promega, Madison, WI, USA). Amplicon libraries were sequenced with a paired-end approach (2 × 250 bp) using the Illumina MiSeq platform (GATC Biotech, Konstanz, Germany).

Data analysis for amplicon sequencing data was performed with QIIME2 version 2019.10 (https://qiime2.org; Bolyen et al. 2019). Primer sequences were removed using cutadapt (Martin 2011). Quality filtering, denoising, and chimeric sequence removal were conducted with the DADA2 algorithm (Callahan et al. 2016). Amplicon sequence variants (ASVs) were then aligned against the reference database Silva v128 (Pruesse et al. 2007) using the VSEARCH classifier (Rognes et al. 2016) to obtain taxonomical information for each ASV. Before statistical analysis, sequencing reads that were assigned to non-target taxa, i.e., unassigned, chloroplasts and mitochondria were removed. The sequencing of 16S rRNA gene fragment amplicons yielded 878,586 prokaryotic high-quality sequences which were clustered into 5,558 ASVs. One sample (Mc1Re, root endosphere of *Matricaria chamomilla*) was not included for further analysis due to a very low number of reads (*n* = 224).

## Functional analysis based on shotgun metagenomic sequencing

Total community DNA aliquots of the four replicate samples (1–4) were pooled together in equimolar ratios. Metagenomic libraries of the bulk soil and all three medicinal plant rhizospheres (*M. chamomilla*, *C. officinalis*, and *S. distichum*) were constructed and sequenced on the Illumina HiSeq platform (2 × 150 bp; 2 × 250 bp) (Eurofins Genomics, Ebersberg, Germany; Washington State University, College of Medical Sciences, Spokane, WA, USA) and the Illumina MiSeq platform (2 × 250 bp) (Lucigen, Middleton, WI, USA).

Removal of Illumina adaptor and quality filtering were performed using Trimmomatic (Bolger et al. 2014). The high-quality reads were *de novo* assembled using MEGAHIT v1.2.9 with meta-sensitive parameters (Li et al. 2015). Only contigs with a length > 1 kb were retained for subsequent analyses. Open reading frames of the assembled metagenome contigs were predicted using Prodigal v2.6.3 (Hyatt et al. 2010). To remove redundant sequences, CD-HIT-EST was used to cluster protein-coding gene sequences into a non-redundant gene catalog using a nucleotide identity of 95% similarity (Li and Godzik 2006). Non-redundant genes were further annotated using the eggNOG-mapper and the blast algorithm in DIAMOND (Buchfink et al. 2015; Huerta-Cepas et al. 2017) and the eggNOG database (Huerta-Cepas et al. 2019). Quantifications were accomplished by mapping high-quality reads to assembled contig annotations using BWA (Li and Durbin 2010), then parsed using featureCounts of the Subread package (Liao et al. 2014).

## Reconstruction of bacterial metagenome-assembled genomes

Metagenome-assembled genomes (MAGs) were constructed from shotgun metagenome datasets using a combination of different binning methods, namely Maxbin2 MetaBAT2 and CONCOCT (Alneberg et al. 2014; Kang et al. 2019; Wu et al. 2016). High-quality genomes were selected using DASTool (Sieber et al. 2018) and then dereplicated using dRep v2.2.3 (Olm et al. 2017) to obtain a non-redundant metagenome-assembled bacterial genome set. The quality of MAGs was estimated using CheckM (Parks et al. 2015). Only medium-quality MAGs according to the current definition of the minimum information metagenome-assembled genome (MIMAG) standards (Bowers et al. 2017) were kept for further analyses. Taxonomical information for each metagenome-assembled genome was obtained using GTDB-Tk (Chaumeil et al. 2019). Prediction of protein-coding sequences and gene annotations were performed using DRAM (Shaffer et al. 2020). Abundance profiles for each MAG were calculated by using CoverM with the option -m rpkm, --min-read-aligned-percent 0.75, and --min-covered-fraction 0.

### Statistical analysis

The statistical analyses were conducted with RStudio v1.3.1093 using Phyloseq, and vegan R packages (R Core Team 2013; McMurdie and Holmes 2013; Oksanen et al. 2007). Bacterial datasets were normalized by rarefying to the lowest number of reads and using MetagenomeSeq’s cumulative sum scaling (CSS; (Paulson et al. 2013) for subsequent alpha (bacterial richness and diversity) and beta (variation in bacterial composition) diversity analysis. The non-parametric (ranked-based) Kruskal-Wallis test was used to statistically examine differences in alpha diversity (number of observed ASV and Shannon diversity index) between compartment and plant species. Bacterial community profiles were used to generate a Bray-Curtis matrix distance and visualized using a PCoA plot. The Bray-Curtis matrix distance was then subjected to PERMANOVA using Adonis to test for significant effects of the compartment and plant species on the bacterial community structures. Bacterial taxa that enriched the tested factors were identified using a linear discriminant analysis effect size (LefSe) (Segata et al. 2011). The Bray-Curtis dissimilarity matrix was created using gene profiles from both bulk soil and the rhizosphere of three medicinal plants. This matrix was subsequently utilized to perform hierarchical clustering.

## Results

### Compartment was the major factor influencing bacterial richness and diversity

**Fig. 1:**
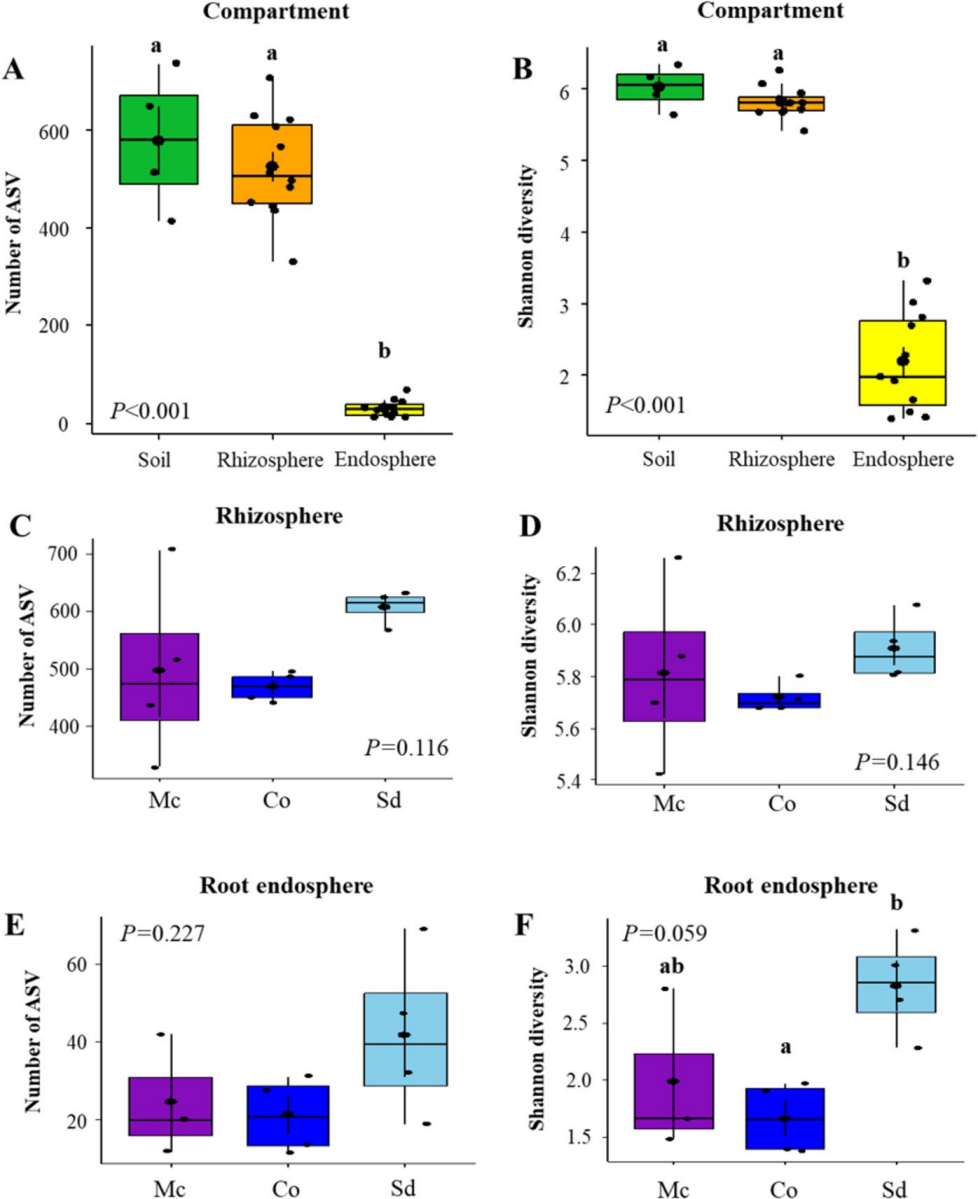
Bacterial diversity in bulk soil, rhizosphere, and root endosphere of German chamomile (Mc -***Matricaria chamomilla*** L.), marigold (Co -***Calendula officinalis*** L.), and night shade (Sd -***Solanum distichum*** Schumach. and Thonn.). The comparison of bacterial diversity is based on the number of ASVs (**a**) and the Shannon diversity index (**b**) in different compartments. The comparison of bacterial diversity is based on the number of ASVs (**c**) and the Shannon diversity index (**d**) in the rhizosphere of different host plants. The comparison of bacterial diversity is based on the number of ASVs (**e**) and the Shannon diversity index (**f**) in the root endosphere of different host plants. The dots in the box plots represent the number of ASVs (A, C, and E) and the Shannon diversity index (B, D, and F) for each sample

The highest bacterial richness (number of observed ASVs - n_ASV_) and diversity (Shannon diversity index - H’) were observed in soil samples (n_ASV_ = 578 and H’ = 6.0, Fig. 1A and B). Rhizosphere samples exhibited slightly lower values (n_ASV_ = 524 and H’ = 5.8), with an average taken from the three plant species, followed by root endosphere samples (n_ASV_ = 29.6 and H’ = 2.2). A high number of shared ASVs was detected in rhizosphere and soil samples (*C. officinalis* – 43.9% − 431 ASVs, *M. chamomilla* – 46.9% − 522 ASVs, and *S. distichum* – 24.1% − 330 ASVs, Supplementary Figure S1). When statistically assessed using the Kruskal-Wallis test and followed by a pairwise Wilcox test, soil, and rhizosphere had higher bacterial richness and diversity in comparison to root endosphere samples (Fig. 1A and B, *P* < 0.001). When the effect of plant species on bacterial richness and diversity was investigated, we observed that bacterial richness and diversity in rhizosphere samples of *S. distichum* were relatively higher (n_ASV_ = 606 and H’ = 5.9) than in the other plant species (*M. chamomilla* - n_ASV_ = 496 and H’ = 5.8; *C. officinalis* - n_ASV_ = 469 and H’ = 5.7, Fig. 1C and D). However, there were no significant differences in bacterial richness and diversity between different plant species in the rhizosphere according to the Kruskal-Wallis test. Interestingly, the plant species showed a weak effect (*P* = 0.059, Fig. 1E) on bacterial diversity but did not influence bacterial richness in the root endosphere (*P* = 0.227, Fig. 1D). Pairwise comparison indicated a higher bacterial diversity in the root endosphere of *S. distichum* (H’ = 2.8) in comparison to other plant species (*M. chamomilla* - n_ASV_ = 496 and H’ = 1.9; *C. officinalis* - n_ASV_ = 469 and H’ = 1.7).

A higher impact of plant species on the rhizosphere bacterial community in comparison to the root endosphere bacterial community was observed.

**Fig. 2:**
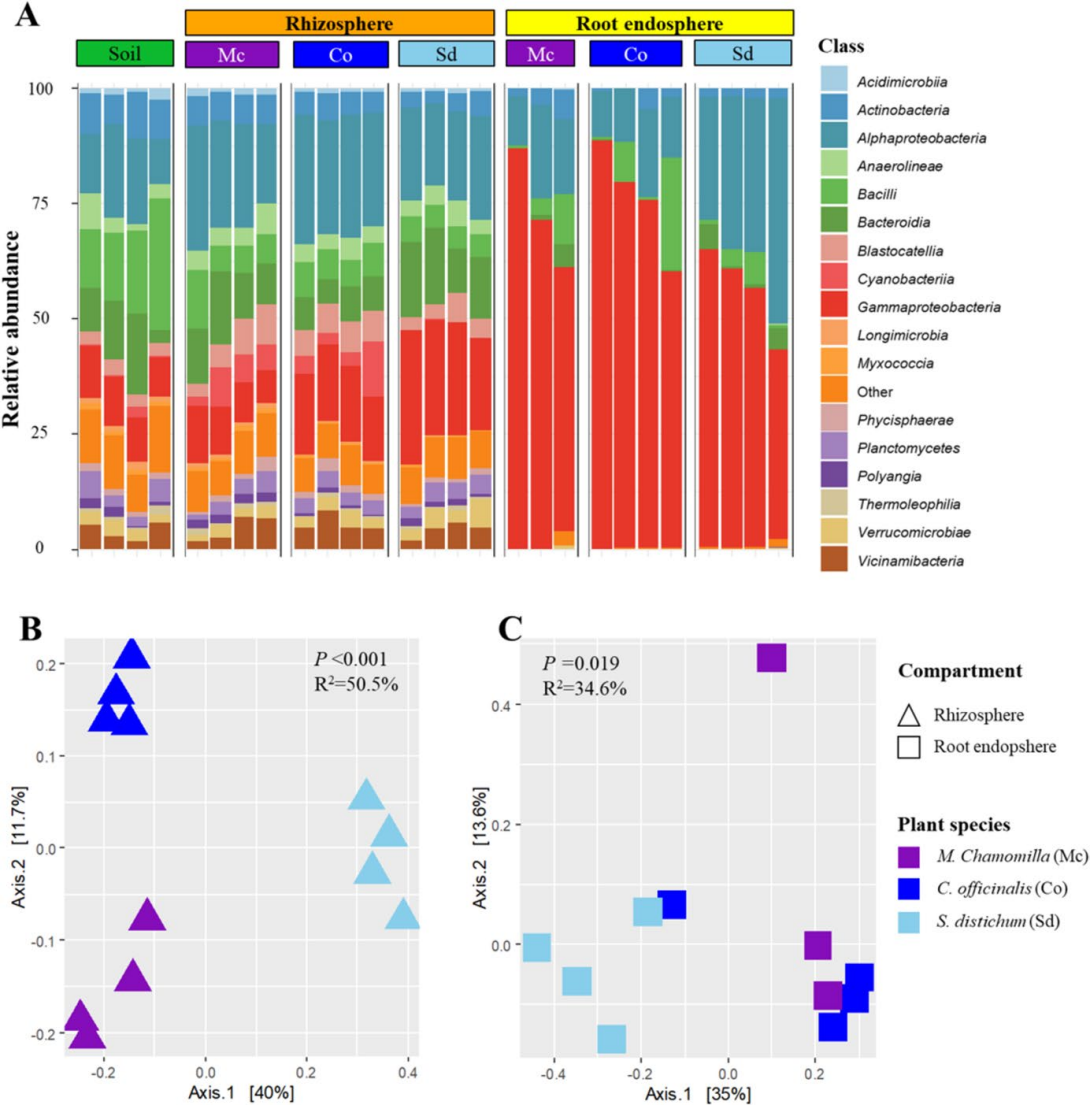
Bacterial community composition and structure in bulk soil, rhizosphere, and root endosphere of German chamomile (Mc: ***Matricaria chamomilla*** L.), marigold (Co: ***Calendula officinalis*** L.), and night shade (Sd: ***Solanum distichum*** Schumach. and Thonn.). The bacterial community composition is shown at the class level (**a**). Bacterial community clustering in the rhizosphere (**b**) and root endosphere (**c**) were visualized in a two-dimensional PCoA plot based on a Bray–Curtis matrix. Different shapes represent plant compartments, while the colors indicate the plant species

Four bacterial classes, *Alphaproteobacteria*, *Bacilli*, *Bacteroidia*, and *Gammaproteobacteria*, were identified as the dominant taxa in all compartments and contributed to 54.8, 57.0, and 97% of total reads in soil, rhizosphere, and root endosphere samples, respectively (Fig. 2A). When soil and rhizosphere samples were compared, we observed that the relative abundance of *Alphaproteobacteria* (rhizosphere − 22.9% vs. soil – 15.4%) was higher in the rhizosphere whereas the relative abundance of *Actinobacteria* and *Bacilli* was higher in soil. Moreover, the relative abundance of the bacterial order *Rhizobiales* (*Alphaproteobacteria*) was higher in the rhizosphere, where it ranged between 6.6 and 8.8% in comparison to 4.9% in soil (Supplementary Figure S2A). The abundance of this group increased in the root endosphere, where it ranged between 12.8 and 21.8%. *Gammaproteobacteria*, which are composed of two dominant bacterial taxa i.e., *Herbaspirillum* and *Pseudomonas*, were highly prevalent in the root endosphere (68.5%, Supplementary Figure S2B) in comparison to soil (10.3%) and rhizosphere samples (16.8%). In contrast, *Bacteroidia* showed the opposite pattern as this group found in low abundance in the root endosphere (1.5%) in comparison to soil (10.6%) and the rhizosphere (10.8%).

### The compartment and plant species affected bacterial community structures

Beta diversity analysis revealed significantly different clustering between bacterial community structures of soil, rhizosphere, and root endosphere samples (*P* < 0.001, R^2^ = 45.2%, Supplementary Figure S3). Rhizosphere samples from *M. chamomilla* and *C. officinalis* showed a tendency to cluster with bulk soil, suggesting a greater similarity in their community composition when compared to the rhizosphere samples from *S. distichum* (Supplementary Figure S3). Three separated clusters according to plant species were observed for the rhizosphere samples (Fig. 2B). Interestingly, the root endosphere samples of *M. chamomilla* and *C. officinalis* clustered together whereas samples of *S. distichum* were separated from this cluster. The plant species explained 50.5% of the variation in bacterial community structures in the rhizosphere and 34.6% of the variation in the root endosphere (Fig. 2B and C). Our results indicate a stronger effect of the plant species in terms of modulating bacterial community structures in rhizosphere samples in comparison to root endosphere samples. To assess which rhizosphere bacterial genera differed between the plant species, a linear discriminant analysis effect size analysis was performed. Of the 715 detected bacterial genera, a total of 22 bacterial genera were found to be differentially abundant between plant species (Supplementary Table S1). A higher number of ASVs were enriched in *S. distichum* (*n* = 14) in comparison to *M. chamomilla* (*n* = 3) and *C. officinalis* (*n* = 5). Interestingly, two bacterial genera, *Gemmobacter*,* Acinetobacter* and *Hydrogenophaga*, were only present in the rhizosphere of *S. distichum*, but were not detected in the rhizosphere as well as the root endosphere of *M. chamomilla* and *C. officinalis*.

### A rhizosphere effect at the functional level was observed

Analyses of the prokaryotic community revealed a clear shift in the microbiome composition from bulk desert farm soil to the rhizosphere of medicinal plants cultivated under these conditions. Deepening analyses were implemented to explore if this plant-driven effect is linked to specific biological functions. The metagenomes were comparatively analyzed with a specific focus on overall changes between bulk and rhizosphere soil irrespective of the plant species.

**Fig. 3:**
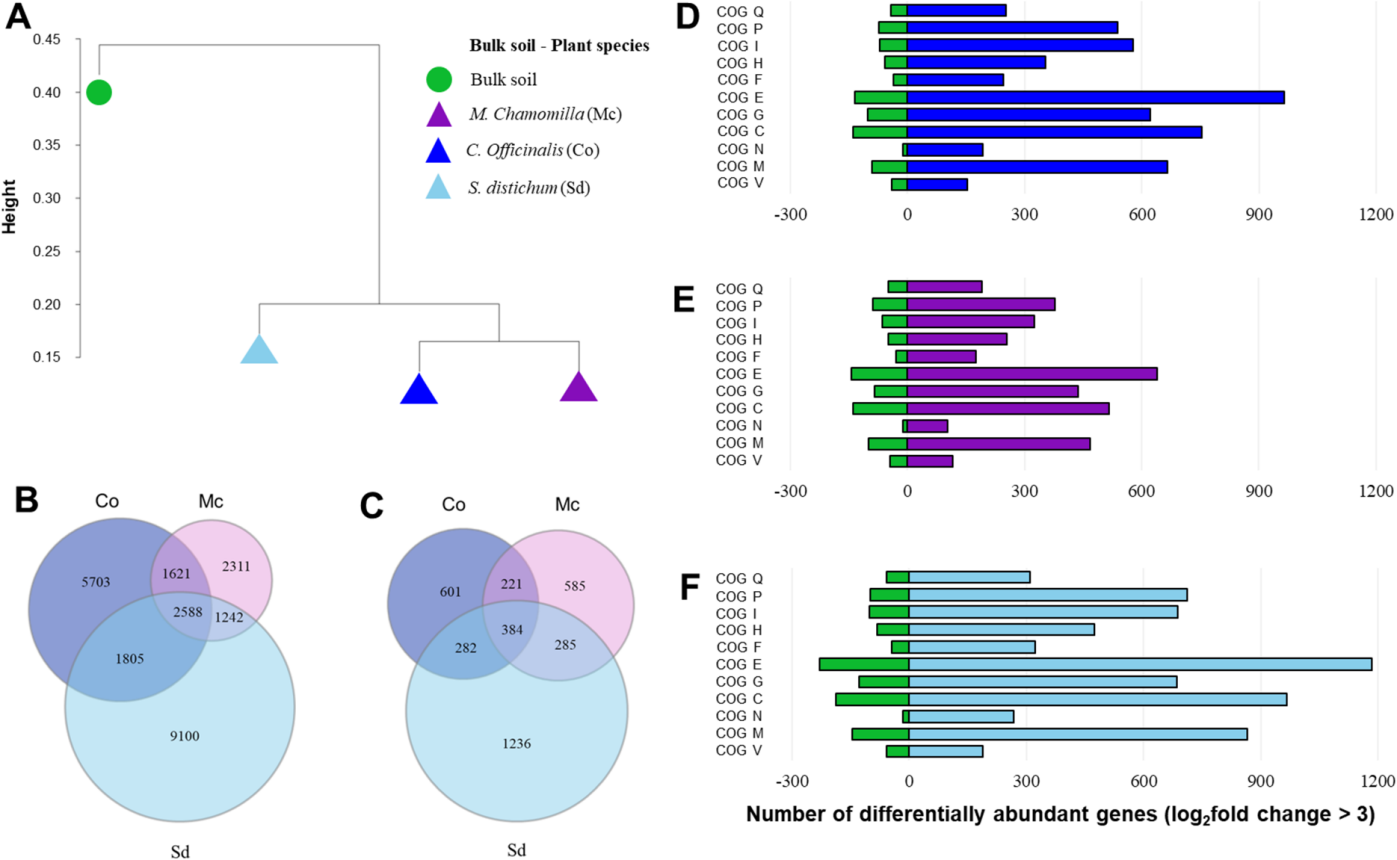
Comparative assessment of functional assignments in bulk soil and rhizosphere metagenomes. Hierarchical clustering of bacterial gene composition between different metagenomic samples is based on a Bray-Curtis dissimilarity matrix (**a**). Number of genes that were enriched (with log_2_fold change > 3) in the rhizosphere metagenomes of each plant species when compared to the bulk soil metagenome (**b**). Number of genes that decreased (with log_2_fold change > 3) in rhizosphere metagenomes of each plant species when compared to the bulk soil metagenome (**c**). Number of differentially abundant genes and their Clusters of Orthologous Group (COG) functional categories in rhizosphere metagenomes of each plant species when compared to the bulk soil metagenome (**d**-**f**). Negative values indicate the number of genes that were enriched in the bulk soil metagenome whereas positive values indicate the number of genes that were enriched in the rhizosphere metagenomes

By analyzing the rarefied dataset, we observed a higher number of annotated genes in bulk soil (*n* = 530,011) in comparison to the rhizosphere of *S. distichum* (*n* = 279,348), *M. chamomilla* (*n* = 279,459) and *C. officinalis* (*n* = 280,588). To analyze the similarities in gene profiles between the bulk soil and the rhizosphere of the three medicinal plants, we constructed hierarchical clustering based on the Bray-Curtis dissimilarity matrix derived from their respective gene profiles. Hierarchical clustering resulted in two major clusters that were divided into rhizosphere of the medicinal plants and bulk soil (Fig. 3A) indicating distinct differences in their microbial functioning on the community level. Following the trend of the bacterial community composition, microbial functioning in the rhizospheres of *M. chamomilla* and *C. officinalis* was more similar in comparison to *S. distichum*.

By using a rarefied gene profile dataset, we detected a total of 24,369 genes (with log_2_fold change > 3) that were enriched in the rhizosphere samples whereas a total of 3594 genes (with log_2_fold change > 3) were decreased when compared to bulk soil samples (Fig. 3B and C). Interestingly, only a small fraction of differentially abundant genes was consistently enriched or decreased by the three plant species (Fig. 3B and C). For instance, only 2588 of 24,369 (10.6%) genes were found to be enriched in all plant species whereas 17,114 genes (70.2%) were only enriched in one plant species. Moreover, only 384 of 3594 (9.1%) genes were detected to be enriched in all plant species, whereas 2422 genes (62.2%) were only decreased in one plant species. Within KEGG functional categories, enrichment in the rhizospheres in comparison to bulk soil was found for the categories amino acid ion transport as well as metabolism, energy production and conversation, and carbohydrate transport and metabolism. Furthermore, enrichment from bulk soil in all three plant rhizospheres was found for the categories representing defense mechanisms and secondary metabolites biosynthesis. These results indicated that different plant hosts specifically modulated microbial function in the rhizosphere.

A deepening analysis indicated that the rhizosphere in comparison to bulk soils had a higher number of enriched genes encoding carbohydrate-active enzymes (CAZymes). In total, 61 different carbohydrate-active enzyme (CAZyme) families were detected in bulk soil and rhizosphere samples. We identified 36 CAZymes families that were enriched in the rhizosphere in comparison to bulk soil, whereas 5 CAZymes families showed the opposite pattern. To explore the ecological context of the CAZymes, predicted genes were grouped according to their potential substrates. In comparison to bulk soil, the rhizosphere had an increased abundance of GHs that are used to break down oligosaccharides and complex polysaccharides, i.e., peptidoglycan, fructan, pectin, chitin, and arabinan. The complex polysaccharides for which degraders decreased in comparison to bulk soil were pectin, starch, alpha-glucan, and glycogen. The positive shifts in the hydrolysis of complex polysaccharides in the rhizosphere were primarily driven by GH105, GH33, and PL11 for pectin, GH19 for chitin, GH29 for glycan, GH32 for fructan, GH103 for peptidoglycan and GH43 and GH51 for arabinan.

### Metagenome-assembled genomes and their contribution to rhizosphere core functions

**Fig. 4:**
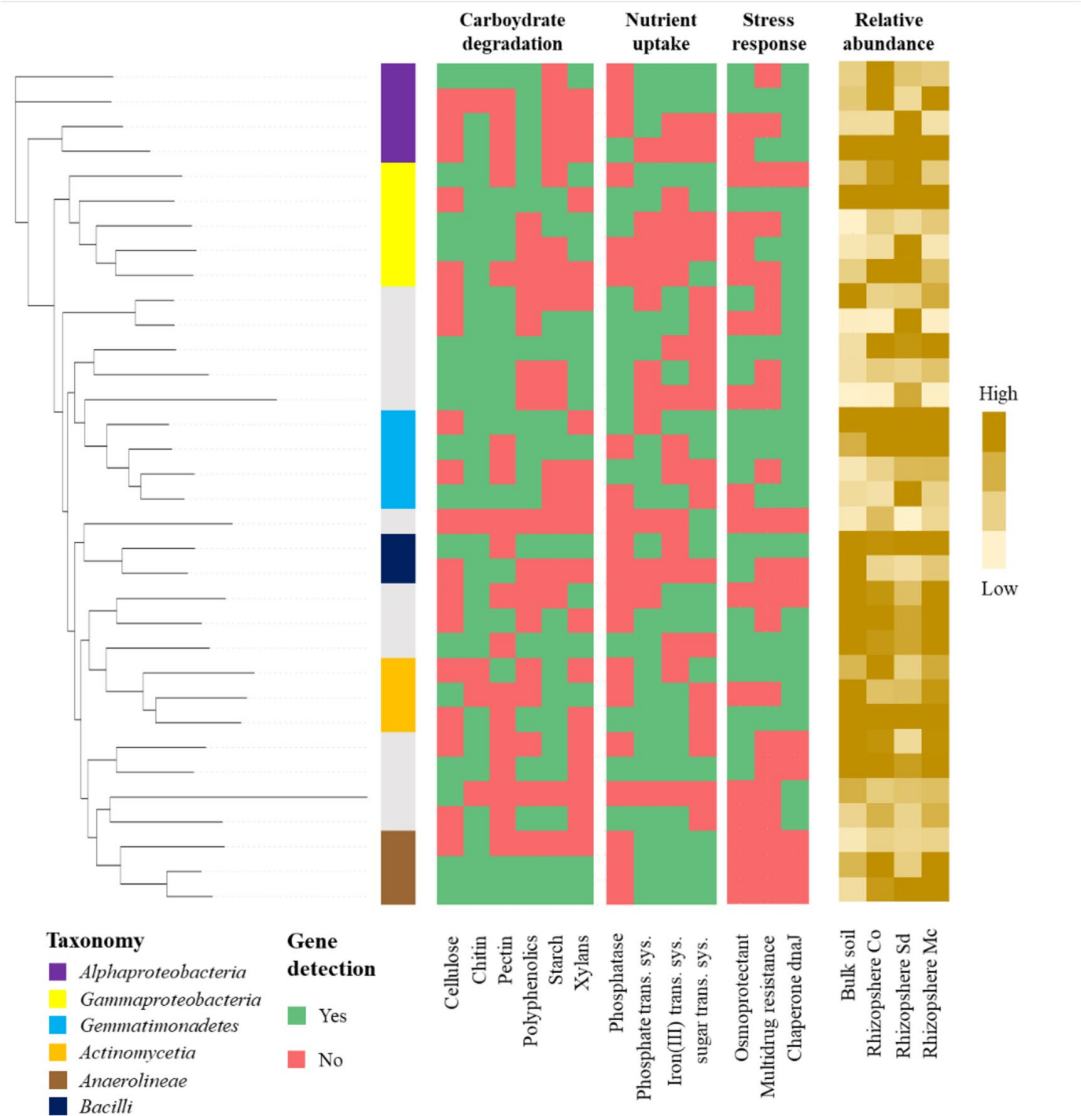
Gene profiles for selected functions in MAGs recovered from bulk soil and rhizosphere samples. Presence (green)/absence (red) plots show profiles of selected genes in MAGs. Relative abundances of MAGs were estimated using coverM based on the number of mapped reads per kilobase per million reads (RPKM)

The binning approach resulted in 34 non-redundant metagenome-assembled genomes (MAGs) with completeness higher than 50% and contamination less than 10%. Within this set, two MAGs were classified as high-quality draft MAGs (completeness higher than 90% and contamination less than 5%). The majority of the MAGs belonged to *Gammaproteobacteria* (*n* = 5), *Alphaproteobacteria* (*n* = 4), *Gemmatimonadetes* (*n* = 4), *Nitrososphaeria* (*n* = 4), and *Anaerolineae* (*n* = 3, Fig. 4).

Both multifunctionally as well as specifically equipped MAGs were found among the plant-specific taxa. *Actinobacteria*, *Anaerolineae*,* Gemmatimonadetes*, *Alpha*- and *Gammaproteobacteria* were found to harbor the functional capability to degrade a range of carbohydrates, particularly chitin, cellulose, pectin, starch, and xylans (Fig. 4). Some of the MAGs could be classified at the genus level, i.e., *Sphingomonas*, *Neorhizobium* (*Alphaproteobacteria*), *Hydrogenophaga*, *Steroidobacter* (*Gammaproteobacteria*), *Pseudarthrobacter*, *Nocardioides* (*Actinobacteria*) and *UBA12294* (*Anaerolineae*). A high number of detected CAZyme was found in *Anaerolineae*, *Gammaproteobacteria*, and *Gemmatimonadetes.* Their MAGs revealed on average 46, 32, and 31 GH-encoding genes per genome, respectively, which is indicative of high activity in oligosaccharide degradation. Interestingly, the relative abundances of the above-mentioned MAGs were higher in rhizosphere samples in comparison to soil samples (Fig. 4). They also harbor the potential to degrade polyphenolic compounds due to the presence of genes that encode laccase, lignin peroxidase, and vanillyl-alcohol oxidase.

## Discussion

By employing a combination of amplicon sequencing and a metagenomics-driven approach, the present study provides new findings highlighting the importance of the plant genotype in rhizosphere microbiome assembly. Here we show that similar microbial functions can be maintained by differing bacterial communities. Plant-beneficial functions within the microbiome at the soil root interface were found to be highly redundant, while other functions associated with carbohydrate and secondary metabolite metabolism were specific. The bacterial diversity in the rhizosphere of the medicinal plants was as high as in bulk soil samples, however, the community composition differed substantially. A major driver was the enrichment of *Rhizobiales* (*Alphaproteobacteria*) in the rhizosphere, which are widely known as plant-beneficial bacteria. A previous study by Köberl et al. (2016) reported that the abundance of *nifD*, *nifH*, and *nifK* genes that are important for nitrogen fixation was highly abundant in the rhizosphere of medicinal plant species in comparison to soil. The soil in most desert areas has low nitrogen and organic matter concentrations (Alsharif et al. 2020). As nitrogen is a crucial macronutrient, the increase of specific microbes that can improve nitrogen availability in the rhizosphere could be related to host plant adaptation to the nitrogen-poor environment. A similar phenomenon was previously observed in hyper-arid environments. Plants can select and accumulate specific bacterial taxa with specific traits, i.e., production of osmoprotectants, auxin, extracellular polymeric substances, and nutrient transport systems that might support the plant hosts to survive under extreme conditions (Marasco et al. 2022; Wicaksono et al. 2022a, b).

Our study demonstrated microbiome differentiation at the rhizosphere soil-root interface and suggests adaptation of rhizosphere and root endosphere microbiome each serving distinct ecological niches. The rhizosphere soil-root interface acts as a selective barrier, limiting endophytic competence to specific bacterial species, and leading to a decrease in diversity in endophytic assemblages. Interestingly, the plant species had a stronger influence on bacterial community structures in the rhizosphere when compared to the root endosphere. The perennial plant *S. distichum* was associated with bacterial communities that showed the most considerable differences to bulk soil from the desert farm in comparison to the other two plant species (Supplementary Figure S3). Different plant life cycles (annual/perennial) were previously shown to have a significant influence on plant microbiome assembly (Samad et al. 2017; Wassermann et al. 2019). This likely results from a longer period during which the host plant can specifically promote bacterial diversity and select a specific microbiome. Furthermore, the bacterial community compositions in the rhizosphere of *M*. *chamomilla* and *C*. *officinalis* were more similar in comparison to *S*. *distichum*. *M*. *chamomilla* and *C*. *officinalis* are phylogenetically closely related (both belong to the *Asteraceae* family). This study provides evidence of a correlation between host phylogeny and microbial community assemblages. Closely related plant species i.e., *M*. *chamomilla* and *C*. *officinalis* likely produce more similar root exudate profiles (Yoneyama et al. 2011) in comparison to *S. distichum*. As root exudates are known to have a strong influence on plant microbiome assembly (Hu et al. 2018; Sasse et al. 2018), a higher similarity of bacterial community structures between the two plants is expected. Interestingly, the root endosphere exhibited less variability in bacterial community structures between the analyzed plant species. The plant innate immune system imposes a significant selection force (Compant et al. 2021) that could decrease bacterial diversity in the root endosphere resulting in a decrease in the variability of bacterial community structures between different plant species. The root endosphere samples were dominated by two bacterial genera, *Herbaspirillum* and *Pseudomonas*. An accumulation of these taxa, which were previously reported as plant growth-promoting bacteria and efficient plant tissue colonizers (Bertani et al. 2016; Cardinale et al. 2008; Hardoim et al. 2015; Straub et al. 2013), suggests that they are of importance for the medicinal plants tested in this study.

Key genes involved in plant-microbe interaction are over-represented in the rhizosphere microbiome. As anticipated, the gene profiles of bulk soil and rhizosphere samples exhibited a comparable trend to that of the bacterial community composition. Furthermore, a higher functional diversity (according to the richness of bacterial protein-coding genes) in the soil when compared to the rhizosphere could be explained by the higher bacterial taxonomic diversity (number of observed bacterial ASVs). Interestingly, specific core functions that are distinctive for rhizosphere communities in desert agroecosystems were observed. Notably, while a distinct clustering of rhizosphere samples from various plants was evident, their gene profiles demonstrated a notable degree of similarity. This observation suggests that plants hosting a specific microbial community may exhibit comparable microbiome functions. The rhizosphere microbiome was characterized by an enrichment of specific genes related to amino acid ion transport and metabolism, carbohydrate transport and metabolism, defense mechanisms, and secondary metabolites biosynthesis. This enrichment could be related to the release of root metabolites that mainly consist of amino acids and carbohydrates and represent important nutrient sources for rhizosphere bacteria (Canarini et al. 2019). Medicinal plants also accumulate secondary metabolites with antimicrobial properties in their roots (Guillon et al. 2006). Therefore, the enrichment of defense mechanisms and secondary metabolite biosynthesis-related genes could be associated with bacterial survival strategies and niche adaptation under antimicrobial pressure (Almeida et al. 2019; Wicaksono et al., 2022a). The study suggests that the rhizosphere undergoes more intimate host-microbe interactions than the bulk soil, with plants influencing the composition of the bacterial community in this area.

Genes encoding enzymes for the degradation of complex polysaccharides are an important feature of rhizosphere colonization. Recent years have seen advancements in our understanding of the biology of root colonization by rhizobacteria (Liu et al. 2024). However, knowledge of the bacterial genetic features responsible for root colonization is still limited. In this study, genes encoding enzymes for the degradation of complex polysaccharides were among the most abundant and diverse classes of enzymes in the rhizosphere. The presence of polysaccharide-degrading enzymes in rhizosphere bacteria might be needed to utilize plant matter as a nutrient source and ensure colonization in the rhizosphere which is a highly competitive environment. For example, genes related to fructan degradation, namely GH32 CAZymes, occurred in higher abundances in the rhizospheres of the *Asteraceae* plants, which are known to accumulate fructan as reserve carbohydrates (Hendry 1993). Bacteria play an important role in the in-situ breakdown of polysaccharides. Interestingly, indigenous bacteria from these medicinal plants also play role in fermentation (Köberl et al. 2019). A recent study showed that rice plants use specific lignin precursors to shape their microbiota (Su et al. 2024). Therefore, the enrichment of genes that are associated with lignin degradation indicates an intricate association between the enriched MAGs and their host plants.

Functions associated with the degradation of polymeric carbohydrates were frequently identified in metagenome-assembled genomes (MAGs) derived from rhizosphere metagenomic samples. Dominant MAGs that were assigned to *Anaerolineae*, *Gammaproteobacteria*, and *Gemmatimonadetes* were multifunctionally equipped with rhizosphere core functions and also harbored a diverse range of encoded carbohydrate-active enzymes (CAZymes) which were aligned to previous studies (Gardner 2016; Xia et al. 2016). In general, the degradation of major plant components is performed by fungi and specific bacteria, i.e., *Gammaproteobacteria* and *Alphaproteobacteria* under aerobic conditions (Dilly et al. 2005; Liu et al. 2015). However, in areas where the oxygen concentration is low, this process requires anaerobic lignocellulose degraders i.e., *Anaerolineae* as the early aerobic breakdown process likely exhausts oxygen (Yan et al. 2018). Overall, the rhizosphere revealed a higher diversity within the carbohydrate-utilizing community over bulk agricultural desert soil. Despite our comprehensive analysis, there are several limitations to note. These include a relatively small number of biological replicates and a limited number of sampling locations. Additionally, soil chemistry factors such as pH, water content, and organic matter content which could influence bacterial community structures, were not addressed in this study. These limitations highlight the need for future research to incorporate a larger sample size and to factor in these relevant elements. Furthermore, the incompleteness of some genomes may restrict the detection of certain genes.

In conclusion, the microbiome continuum at the soil-root interface of different medicinal plant species revealed significant shifts in bacterial community structure, unveiling a high level of plant specificity. This study also identified polysaccharide degradation genes as one of the most abundant classes of enzymes in desert farm soil communities. Overall, the study sheds light on key microbiome constituents involved in rhizosphere functioning.

## Supplementary Information

Below is the link to the electronic supplementary material.

Supplementary file1 (DOCX 542 KB)

## Data Availability

The amplicon sequences are available in the European Nucleotide Archive (www.ebi.ac.uk/ena) under the BioProject accession number PRJEB77103. All other data is publicly available at the Open Science Framework (OSF) repository www.osf.io/qpuh8.
