# Supplementary material for: Plant-specific microbial diversity facilitates functional redundancy at the soil-root interface
Source: Plant Soil. 2024 Dec 5;523(2):811–25. doi: 10.1007/s11104-024-07097-5 (PMC13337600; doi:10.1007/s11104-024-07097-5)
Supplement: Supplementary file 1 — Supplementary file1 (DOCX 542 KB) [file 11104_2024_7097_MOESM1_ESM.docx]

**Plant-specific microbial diversity facilitates functional redundancy at the soil-root interface**

Wisnu Adi Wicaksono*^1*^*, Martina Köberl^1,2*^, Richard Allen White III^3,4^, Janet K. Jansson^2^, Christer Jansson^6^, Tomislav Cernava^1^, Gabriele Berg^1^

*^1^Graz University of Technology, Institute of Environmental Biotechnology, Graz, Austria*

*^2^Pacific Northwest National Laboratory, Earth and Biological Sciences Division, Richland, WA, USA*

*^3^North Carolina Research Campus (NCRC), Department of Bioinformatics and Genomics, The University of North Carolina at Charlotte, Kannapolis, North Carolina, USA*

*^5^Computational Intelligence to Predict Health and Environmental Risks (CIPHER), Department of Bioinformatics and Genomics, The University of North Carolina at Charlotte, Charlotte, North Carolina, USA*

*^6^Pacific Northwest National Laboratory, Environmental Molecular Sciences Laboratory, Richland, WA, USA*

**Supplementary Materials**

**
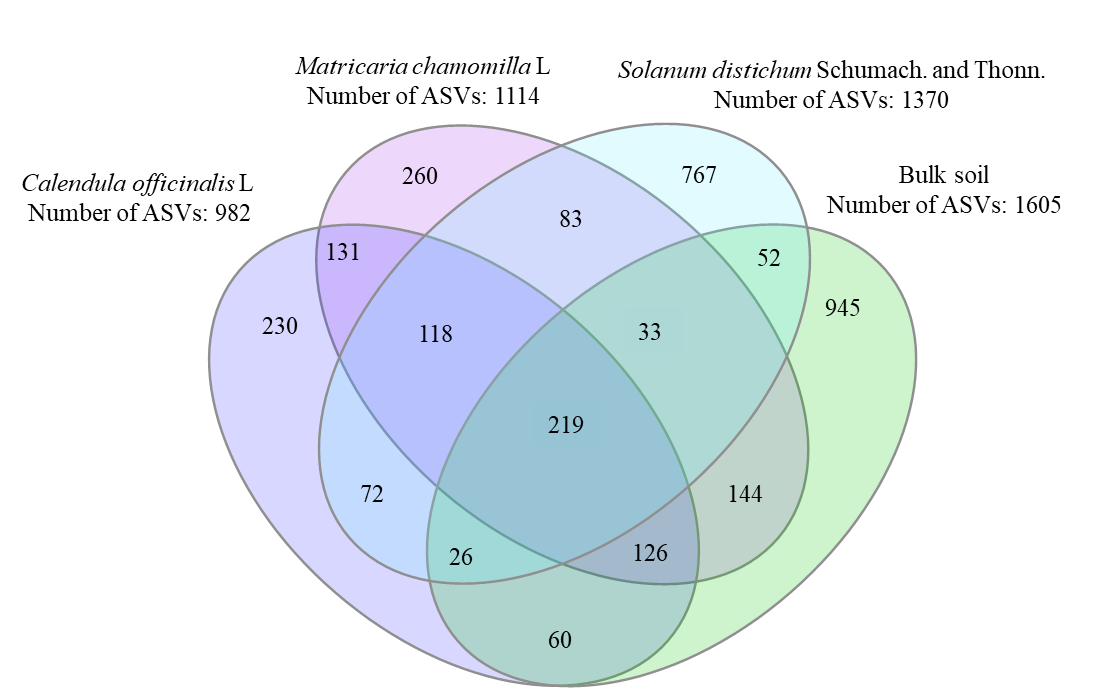
**

**Supplementary Figure S1 Venn diagram showing the unique and shared ASVs between rhizosphere and bulk soil samples.**

**Supplementary
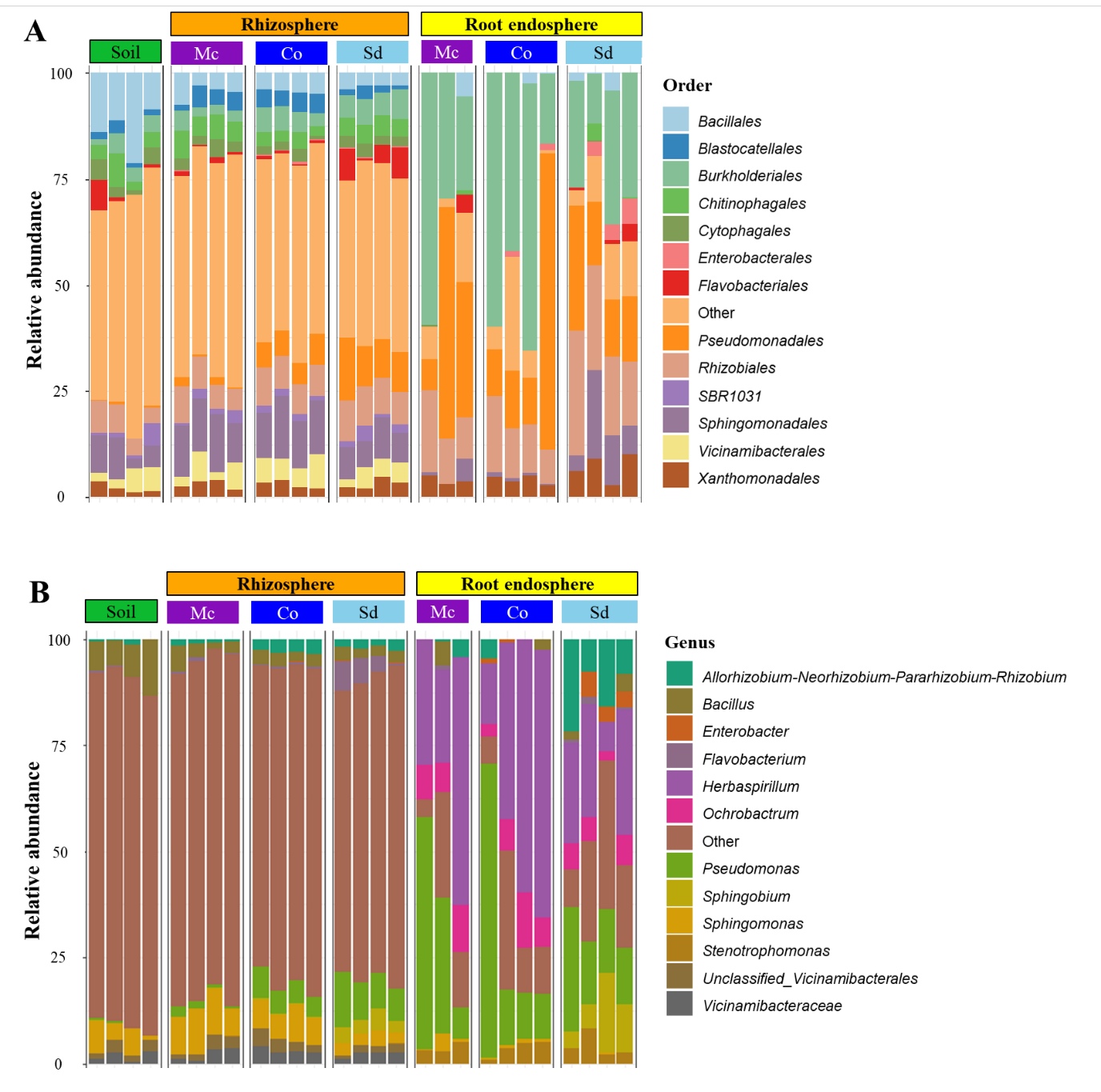
 Figure S2. Bacterial community composition and structure in bulk soil, rhizosphere, and endosphere of chamomile (Mc - *Matricaria chamomilla* L.), marigold (Co - *Calendula officinalis* L.), and night shade (Sd - *Solanum distichum* Schumach. and Thonn.)*.*** The bacterial community composition is shown at the order (**A**) and genus level (B).


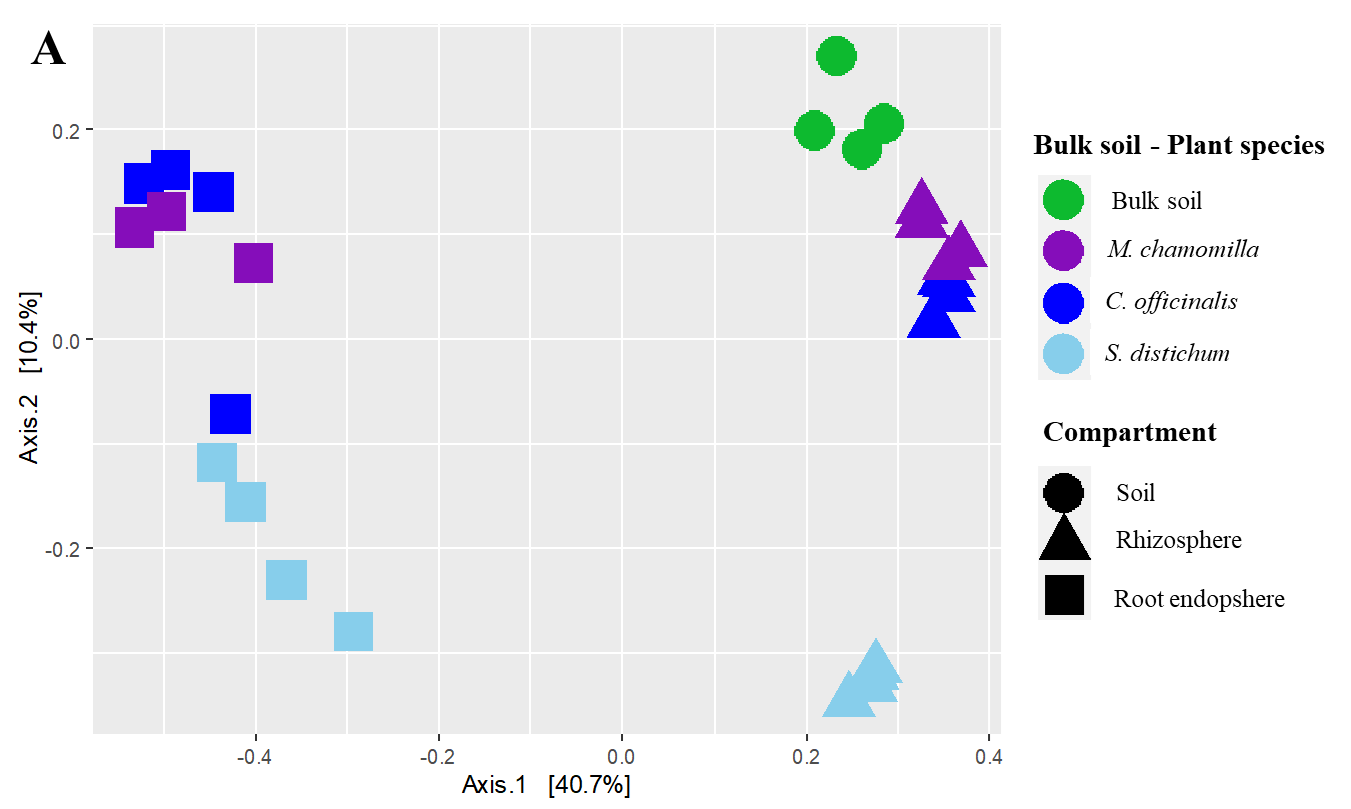


**Supplementary Figure S1. Bacterial community clustering in bulk soil, rhizosphere, and root endosphere was visualized in a two-dimensional PCoA plot based on a Bray–Curtis matrix.**

**Supplementary Table S1 Analysis of significantly enriched bacterial taxa in each plant species by using Linear Discriminant Analysis Effect Size (LEfSe).** This analysis was conducted to determine bacterial biomarkers specific to each plant species at the genus level.


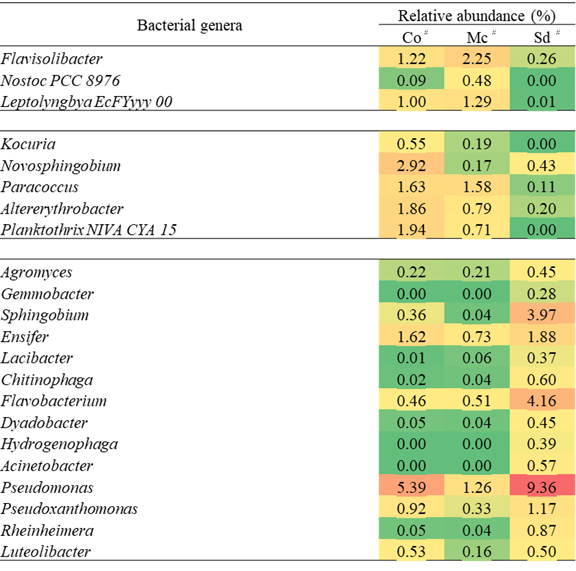


^#^Plant species - German chamomile (Mc - *Matricaria chamomilla* L.), marigold (Co - *Calendula officinalis* L.), and night shade (Sd - *Solanum distichum* Schumach. and Thonn.)*.*
